# Antimicrobial Susceptibility Profiles of *Escherichia coli* Isolates from Clinical Cases of Ducks in Hungary Between 2022 and 2023

**DOI:** 10.3390/antibiotics14050491

**Published:** 2025-05-10

**Authors:** Ádám Kerek, Ábel Szabó, Ákos Jerzsele

**Affiliations:** 1 Department of Pharmacology and Toxicology, University of Veterinary Medicine, István utca 2, HU-1078 Budapest, Hungary; szabo.abel@student.univet.hu (Á.S.); jerzsele.akos@univet.hu (Á.J.); 2 National Laboratory of Infectious Animal Diseases, Antimicrobial Resistance, Veterinary Public Health and Food Chain Safety, University of Veterinary Medicine, István utca 2, HU-1078 Budapest, Hungary

**Keywords:** *Escherichia coli*, antimicrobial resistance, minimum inhibitory concentration, MIC, waterfowl, ducks, MDR

## Abstract

**Background**: Antimicrobial resistance (AMR) poses a growing threat to veterinary medicine and food safety. This study examines *Escherichia coli* antibiotic resistance patterns in ducks, focusing on multidrug-resistant (MDR) strains. Understanding resistance patterns and predicting MDR occurrence are critical for effective intervention strategies. **Methods**: *E. coli* isolates were collected from duck samples across multiple regions. Descriptive statistics and resistance frequency analyses were conducted. A decision tree classifier and a neural network were trained to predict MDR status. Cross-resistance relationships were visualized using graph-based models, and Monte Carlo simulations estimated MDR prevalence variations. **Results**: Monte Carlo simulations estimated an average MDR prevalence of 79.6% (95% CI: 73.1–86.1%). Key predictors in MDR classification models were enrofloxacin, neomycin, amoxicillin, and florfenicol. Strong cross-resistance associations were detected between neomycin and spectinomycin, as well as amoxicillin and doxycycline. **Conclusions**: The high prevalence of MDR strains underscores the urgent need to revise antibiotic usage guidelines in veterinary settings. The effectiveness of predictive models suggests that machine learning tools can aid in the early detection of MDR, contributing to the optimization of treatment strategies and the mitigation of resistance spread. The alarming MDR prevalence in *E. coli* isolates from ducks reinforces the importance of targeted surveillance and antimicrobial stewardship. Predictive models, including decision trees and neural networks, provide valuable insights into resistance trends, while Monte Carlo simulations further validate these findings, emphasizing the need for proactive antimicrobial management.

## 1. Introduction

The discovery of antibiotics revolutionized 20th-century medicine, enabling the effective treatment of the previously fatal infectious bacterial diseases. However, the widespread and often indiscriminate use of antimicrobial agents has accelerated the emergence of resistant bacterial strains, making AMR one of the most pressing issues in healthcare systems today [1]. Antimicrobial resistance (AMR) has emerged as one of the most critical global health challenges and has been recognized by the World Health Organization (WHO) as one of the top ten threats to public health [2]. It is increasingly referred to as a “silent pandemic” [3], due to its deadly but largely unseen effects.

Birds play a crucial role in disseminating and maintaining antimicrobial resistance [4]. In waterfowl farming, AMR presents both a health crisis and a serious economic burden, as antibiotic-resistant bacterial infections contribute to high mortality rates and production losses. Combined with widespread use of antibiotics, conditions in intensive duck farming—such as overcrowding, inadequate hygiene, and environmental stress [5]—facilitate the rapid spread of bacterial infections, increasing the likelihood of resistant strains emerging and persisting [6]. The presence of multidrug-resistant *Escherichia coli* is widespread in the duck industry [7]. Responsible antibiotic use must be accompanied by strict biosecurity measures [8] and pharmacological assessments before antibiotic administration [9].

The increasing prevalence of antibiotic resistance underscores the need to explore alternative approaches to reduce or replace antibiotic use. Potential alternatives include pre- and probiotics [10,11], medium-chain fatty acids [12], plant extracts [13,14,15,16,17,18], antimicrobial peptides [19], and various metal compounds [20]. These strategies are particularly important for the poultry sector [21], which, after the swine industry, is the second-largest consumer of antibiotics in animal agriculture [22].

*E. coli* is a Gram-negative, rod-shaped bacterium and one of the most prevalent facultative anaerobic bacteria commonly found as part of the normal gut microbiota in mammals and birds. However, it is considered a major foodborne pathogen, posing significant risks to agricultural productivity [23], animal welfare, and human health [24,25]. Certain pathotypes can cause severe infections [26], including urinary tract infections, bacteremia, diarrhea, and neonatal meningitis in humans [27,28]. The bacterium is widely used across various industries for enzyme production and serves as an indicator of fecal contamination in food safety assessments [29]. Thus, *E. coli* surveillance is mandatory in livestock and retail meat samples within the European Union [30,31].

Gastrointestinal pathogenic *E. coli* strains (e.g., enteropathogenic *E. coli* (EPEC), enterohemorrhagic *E. coli* (EHEC), can be transmitted through contaminated food or water, leading to severe diarrheal diseases [32,33,34]. Diarrheal diseases remain a significant global health burden, particularly in developing countries, where they not only have the highest incidence rates but also rank among the leading causes of mortality.

Avian pathogenic *E. coli* (APEC), a major subgroup of extraintestinal pathogenic *E. coli* (ExPEC), is responsible for severe respiratory and systemic diseases in poultry, leading to significant economic losses. In poultry health management, respiratory diseases are considered the most economically significant due to their substantial impact on industry losses [35]. APEC causes extraintestinal infections known as colibacillosis, leading to respiratory diseases and cellulitis in poultry [36,37,38]. Colibacillosis in poultry manifests in multiple clinical forms, including airsacculitis, pneumonia, septicemia, pericarditis, perihepatitis, omphalitis, and cellulitis. These conditions result in high mortality, growth retardation, increased medication costs, and carcass condemnation at slaughterhouses, significantly affecting animal welfare and farm profitability [39].

The diarrheagenic *E. coli* (DEC) pathotype is often associated with enteroaggregative *E. coli* (EAEC), a key agent in enteric infections [33,40]. Enteric *E. coli* strains primarily cause watery or bloody diarrhea [41,42].

This study investigates the antimicrobial resistance profiles of *E. coli* isolates obtained from clinical cases and deceased ducks in Hungary between 2022 and 2023. Minimum inhibitory concentration (MIC) determination and phenotypic resistance testing were conducted to assess resistance patterns. Our findings contribute to a deeper understanding of AMR trends and support the development of targeted biosecurity strategies.

## 2. Results

A total of 108 *E. coli* isolates from clinical cases were subjected to phenotypic antimicrobial susceptibility testing, with MIC determination. The majority of isolates originated from Hungary’s Dél-Alföld region (91.7%; *n* = 99), followed by the Dél-Dunántúl region (3.7%; *n* = 4) and the Észak-Alföld region (3.7%; *n* = 4), with a single isolate (0.7%) originating from the Közép-Magyarország region.

**Figure 1:**
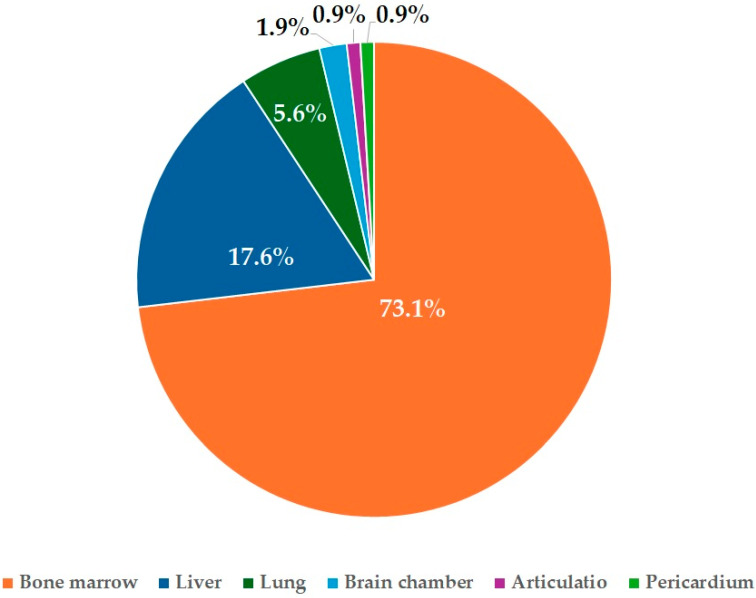
Organ distribution of *Escherichia coli* isolates (*n* = 108) and their relative proportions.

Most isolates (Figure 1) were recovered from samples taken from bone marrow (*n* = 76) or liver (*n* = 19).

**Figure 2:**
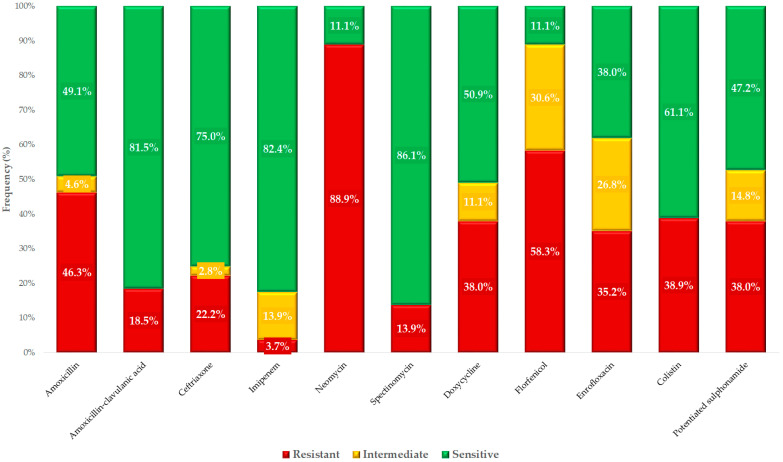
Phenotypic antimicrobial susceptibility profile of *Escherichia coli* isolates (*n* = 108) from ducks, tested against clinically relevant antimicrobials in veterinary and public health contexts.

Based on clinical breakpoints, we determined the proportion of isolates classified as susceptible, intermediate, or resistant to each tested antimicrobial agent (Figure 2). The majority of isolates (88.9%) were resistant to neomycin, while concerning levels of resistance were also observed for florfenicol (58.3%), colistin (38.9%), and enrofloxacin (35.2%). The lowest resistance rate was detected for imipenem (3.7%), although 13.9% of isolates exhibited reduced susceptibility.

**Figure 3:**
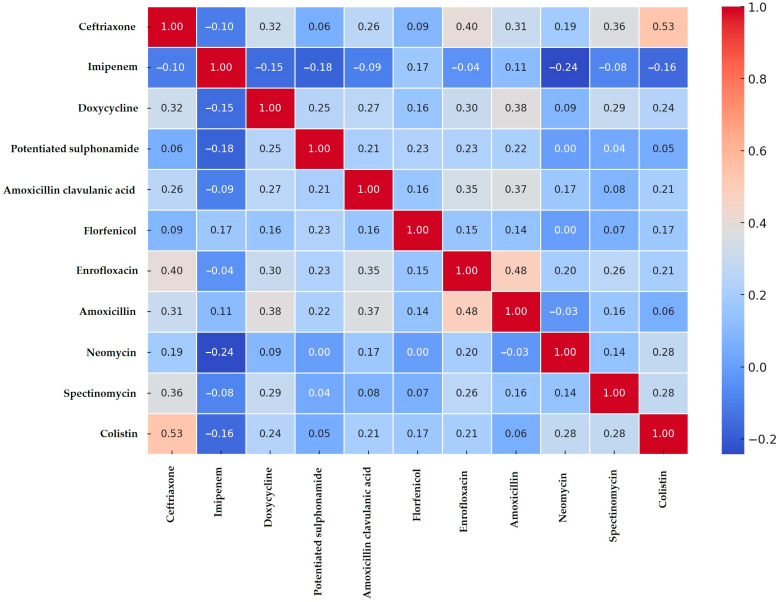
Correlation analysis of antimicrobial resistance among *Escherichia coli* isolates, visualized as a heatmap for each antimicrobial.

Correlation analysis was performed to identify associations between resistance patterns among antibiotics based on clinical breakpoint-derived resistance data (Figure 3). Strong positive correlations were observed between ceftriaxone and colistin (0.53), amoxicillin and doxycycline (0.38), and enrofloxacin and amoxicillin-clavulanic acid (0.35). A notable negative correlation was found between imipenem and neomycin (−0.24).

We assessed the prevalence of multidrug-resistant (MDR) strains, finding that 79.6% (*n* = 86) of isolates were MDR, defined as resistant to at least three tested antibiotics. Extensively drug-resistant (XDR) strains accounted for 28.7% (*n* = 31), while no pan-drug-resistant (PDR) isolates were identified.

The MIC frequency distributions for each antimicrobial agent are summarized in Table 1. For agents lacking established clinical breakpoints, MIC distributions are presented in Supplementary Table S1. Based on clinical breakpoints, approximately half of the tested population remained susceptible to amoxicillin, amoxicillin-clavulanic acid, ceftriaxone, imipenem, spectinomycin, doxycycline, enrofloxacin, and colistin. However, 90% of isolates exhibited resistance to at least one tested antibiotic.

When applying the epidemiological cut-off values (ECOFF) defined by the European Committee on Antimicrobial Susceptibility Testing (EUCAST), approximately half of the tested population was classified as wild-type for doxycycline and colistin. The proportion of non-wild-type strains for each antimicrobial is presented in Supplementary Figure S1.

Detailed MIC values and additional isolate-specific information are available in the Supplementary Materials.

**Figure 4:**
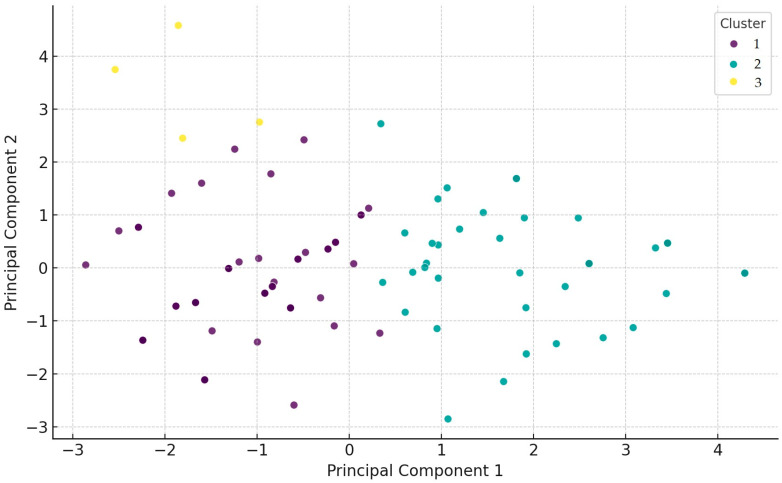
Principal components analysis (PCA) based on resistance patterns identified three major clusters. Isolates in Cluster 1 are marked in purple, Cluster 2 in green, and Cluster 3 in yellow.

Principal components analysis (PCA) was performed based on resistance patterns (Figure 4). Three major clusters were identified. Isolates in Cluster 1 (purple) exhibited high resistance to neomycin (86%) and potentiated sulfonamides (78%). Cluster 2 (green) was characterized by high resistance to neomycin (98%), amoxicillin (80%), and enrofloxacin (75%). Cluster 3 (yellow) consisted of isolates resistant to imipenem.

**Figure 5:**
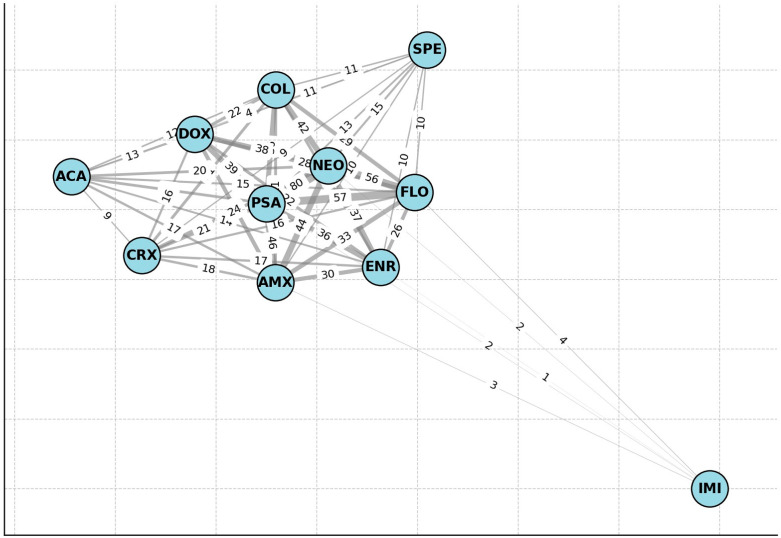
Network analysis of resistance patterns using graph-based models. Imipenem-resistant isolates formed a distinct group. ACA—amoxicillin clavulanic acid; CRX—ceftriaxone; DOX—doxycycline; COL—colistin; PSA—potentiated sulphonamide; SPE—spectinomycin; NEO—neomycin; FLO—florfenicol; AMX—amoxicillin; ENR—enrofloxacin; IMI—imipenem.

Network analysis was conducted using graph-based models (Figure 5), which revealed that resistance to neomycin and spectinomycin frequently co-occurred. Additionally, the resistance to doxycycline, amoxicillin, and florfenicol was commonly observed together. Isolates resistant to imipenem formed a distinct subgroup. The strongest association with other antibiotics was found for potentiated sulfonamides.

**Figure 6:**
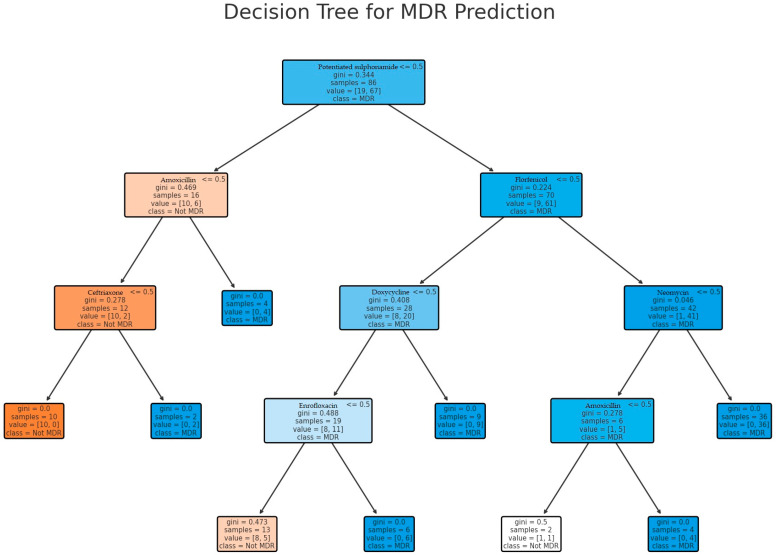
Decision tree model for predicting MDR strain occurrence. Potentiated sulfonamide resistance was selected as the starting point due to its strong association with other antimicrobials.

A predictive model was subsequently developed (Figure 6) to classify MDR strains. Potentiated sulfonamides were selected as the primary feature, as network analysis indicated the strongest correlation with other antibiotics. The model achieved 100% accuracy (precision, recall, and F1-score) in classifying MDR strains. The most influential branching points in the model were antibiotics that strongly determined MDR status. This suggests that resistance to potentiated sulfonamides significantly influenced whether isolates were also resistant to florfenicol, doxycycline, and enrofloxacin or to florfenicol, neomycin, and amoxicillin.

**Figure 7:**
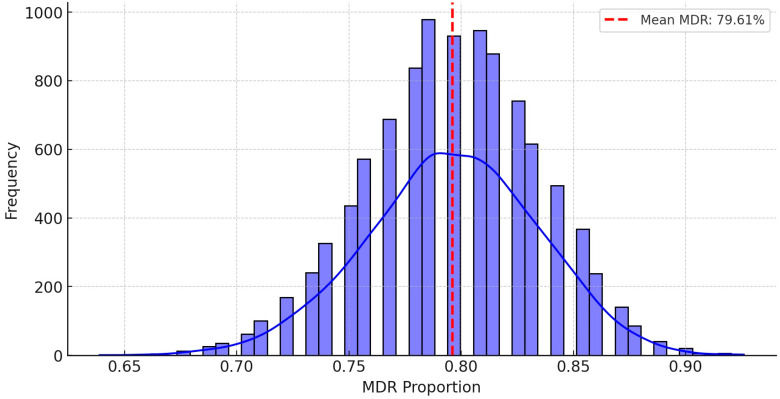
Monte Carlo simulation-based stochastic modeling to predict MDR strain prevalence.

To further estimate MDR prevalence under different antibiotic usage scenarios, we performed stochastic modeling using a Monte Carlo simulation (Figure 7). This approach enables the prediction of potential MDR prevalence rates through random sampling across thousands of iterations, illustrating the probability of an increase or decrease in MDR frequency. The simulation estimated an average MDR prevalence of 79.6%, with the mean and median coinciding, indicating a symmetrical distribution. The standard deviation was 3.86%, with MDR prevalence typically ranging between 75% and 84%. The 95% confidence interval for MDR prevalence was 73.1–86.1%.

**Figure 8:**
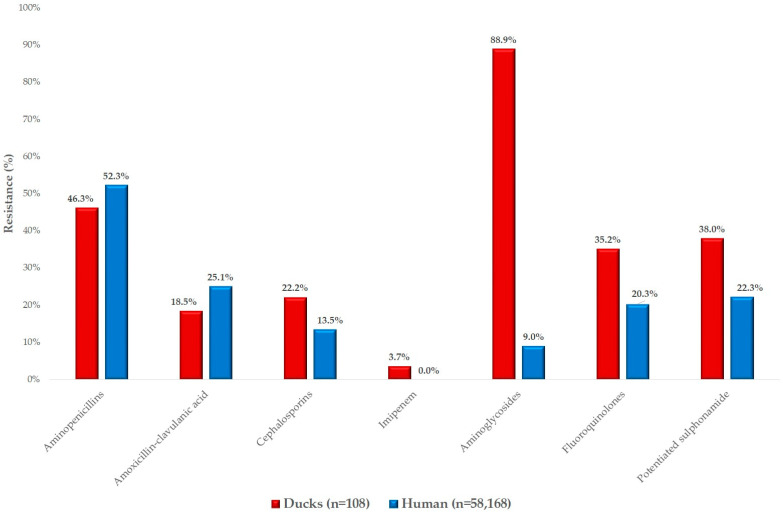
Comparison of *Escherichia coli* resistance rates in duck isolates with human resistance data provided by the National Public Health and Pharmaceutical Center.

Finally, we compared our findings with human resistance data (Figure 8). Resistance patterns were highly similar for aminopenicillins in both human and animal isolates. However, resistance to cephalosporins was higher in veterinary isolates (22.2%) compared to human clinical isolates (13.5%). In contrast, resistance to aminoglycosides was significantly more prevalent in veterinary isolates.

## 3. Discussion

A total of 108 *E. coli* isolates from clinical cases with fatal outcomes were subjected to antimicrobial susceptibility testing over a one-year period (2022–2023). The majority of isolates (91.7%) originated from the Dél-Alföld region of Hungary, reflecting the geographical concentration of the waterfowl industry in this area. This regional concentration is largely influenced by historical factors, such as the longstanding tradition of intensive waterfowl farming, and environmental conditions, including the availability of extensive wetland habitats. Additionally, specific farming practices characterized by high stocking densities and regionally variable antibiotic usage policies may contribute to the observed AMR patterns. These findings emphasize the importance of considering not only geographical but also management-related factors when investigating antimicrobial resistance trends.

*E. coli* is widely used as an indicator species in AMR studies [30,43,44] and plays a particularly important role in waterfowl farming, where antibiotic use is significant. Our findings revealed that over 70% of the isolates were resistant to neomycin, while resistance to florfenicol exceeded 50%.

Resistance levels varied significantly compared to previously published data. For instance, the amoxicillin resistance rate in our study was 46.3%, whereas Afayibo et al. (2022) reported 84% [45]. These discrepancies may stem from differences in geographical factors, antibiotic usage practices, and testing methodologies. The strong positive correlation between amoxicillin and doxycycline resistance (r = 0.38) suggests that these antibiotics are frequently used together or sequentially, potentially promoting cross-resistance. Although differences in authorization status exist among countries, both amoxicillin and doxycycline remain approved for veterinary use in Hungary, under regulated conditions ensuring food safety.

Similarly, the ceftriaxone resistance rate in our study was 22.2%, while previous studies reported 29% [45] and 11.4% resistance [46]. However, Varga et al. [47] and Jeong et al. [48] did not detect resistant strains. It is important to note that the use of ceftriaxone is currently prohibited in veterinary medicine in the European Union, and resistance patterns observed likely reflect past exposures or environmental contamination. The strong correlation between ceftriaxone and colistin resistance (r = 0.53) suggests that resistance to these antibiotics may be co-selected through the presence of linked resistance genes on mobile genetic elements, rather than through direct antibiotic exposure, considering that colistin does not efficiently cross the intestinal barrier [49]. Resistance to third-generation cephalosporins, such as ceftriaxone, is typically associated with the production of extended-spectrum beta-lactamases (ESBLs). Standard testing methods, including the Jarlier et al. (1988) procedure [50] and the double disk synergy test, are recommended to confirm ESBL production. However, in this study, resistance was assessed based on MIC values without specific ESBL confirmatory testing, which represents a limitation of the present work.

Resistance to amoxicillin-clavulanic acid was 18.5% in our isolates, whereas Yassin et al. [46], Varga et al. [47], and Jeong et al. [48] did not detect resistant strains. This may be attributed to the presence of clavulanic acid, which can maintain antibiotic efficacy in some *E. coli* strains. However, given the high prevalence of *β*-lactamase-producing *E. coli*, it is also possible that resistance mechanisms rendering this combination ineffective have already emerged in certain populations [51].

Resistance to imipenem, a carbapenem antibiotic, was detected in 3.7% of the isolates we tested, while other studies reported no resistant strains [45]. The negative correlation between imipenem and neomycin resistance (r = −0.24) suggests that these antibiotics exert selective pressure in different ways and do not exhibit clear cross-resistance. These variations likely reflect differences in antibiotic use policies and regional variations in veterinary administration of carbapenems.

Network analysis highlighted the complexity of AMR patterns. Strong associations were observed between neomycin and spectinomycin resistance, as well as between amoxicillin and doxycycline resistance. This suggests that cross-resistance between aminoglycosides and tetracyclines is widespread, which has significant implications for antibiotic stewardship. Decision tree models and neural network predictions confirmed that enrofloxacin, neomycin, and amoxicillin-clavulanic acid were the most significant predictors of MDR status. The predictive models demonstrated that machine learning tools can effectively identify MDR strains, contributing to better management of antimicrobial resistance.

Doxycycline resistance was detected in 38% of duck isolates, whereas Cen et al. reported a much higher rate of 95.4% [52]. This discrepancy suggests that doxycycline usage varies significantly across geographical regions, and the spectrum of antimicrobial agents used in duck farming differs between countries.

Florfenicol resistance was observed in 58.3% of isolates, which is consistent with the 62% resistance rate reported by Afayibo et al. [45]. This suggests that florfenicol resistance may be widespread in *E. coli* strains isolated from ducks, likely as a consequence of extensive or prolonged usage.

For enrofloxacin, we recorded a resistance rate of 35.2%, while Afayibo et al. reported 100% in 2022 [45], and Jeong et al. found 58.6% in 2021 [48]. In 2017, Yassin et al., however, did not detect any resistant strains [46]. The wide variability in enrofloxacin resistance highlights differences in fluoroquinolone use across regions and emphasizes the role of national regulatory policies in shaping resistance trends.

Colistin resistance was detected in 38.9% of isolates, whereas Jeong et al. [48]. found no resistant strains in ducks. Moreover, antibiotic usage, including colistin, may differ substantially between farms and regions due to several factors. Local farming practices are often shaped by the information accessible to farmers and their level of education. Additionally, the availability of specific antibiotics is frequently influenced by factors such as cost and regulatory constraints. Furthermore, husbandry practices significantly impact bird health, the spread of infections, and the necessity for specific antibiotics. Collectively, these factors determine the extent of antimicrobial exposure faced by *E. coli* strains, thereby influencing their potential to develop resistance.

For potentiated sulfonamides, we observed a resistance rate of 38.0%, compared to 97.7% reported by Yassin et al. [46], 51.7% by Jeong et al. [48], and only 16.7% by Varga et al. [47]. These substantial variations likely reflect differences in sulfonamide use across regions and industries, leading to significant fluctuations in resistance patterns.

Monte Carlo simulation results estimated an average MDR prevalence of 79.6%, with a 95% confidence interval ranging from 73.1% to 86.1%. These findings further validate the high prevalence of MDR strains and underscore the need for continuous AMR surveillance. The observed high prevalence of MDR isolates highlights a significant risk for treatment failures in veterinary practice and raises concerns regarding the potential transmission of resistant strains to humans through the food chain. Cross-resistance correlations among antibiotics may be explained by the co-selection of resistance genes located on mobile genetic elements, such as plasmids and integrons. Although the Monte Carlo simulations provide valuable statistical estimates, the practical implications include the urgent necessity for stricter antibiotic stewardship programs, regular resistance monitoring, and the implementation of targeted biosecurity measures in poultry production systems.

AMR represents a major global public health threat, jeopardizing the ability to combat bacterial infections, as demonstrated by the emergence of multidrug-resistant “superbugs” [53,54]. Our findings support the importance of the WHO’s One Health approach, which emphasizes the interconnected health of animals, humans, and the environment [55,56]. The extensive use of antibiotics in intensive duck farming not only affects livestock but also has environmental and indirect human health implications [57,58]. The widespread application of antimicrobial agents contributes to the dissemination of resistant bacteria [59,60], posing a significant public health risk [61,62]. Veterinarians play a crucial role in monitoring antibiotic use [63,64], ensuring responsible administration, and minimizing the emergence of resistant strains [65,66].

The zoonotic potential of antimicrobial-resistant *E. coli* strains originating from ducks cannot be overlooked. Although direct evidence of transmission is limited, the contamination of meat products, environmental runoff, and occupational exposure represent possible pathways for the transfer of resistant bacteria from waterfowl to humans. These risks underline the importance of comprehensive surveillance programs across the food production chain [67,68].

Overall, our study highlights the need for a multidisciplinary approach to combat antimicrobial resistance, integrating epidemiological surveillance, machine learning-based prediction models, and responsible antibiotic usage strategies. In Hungary, veterinary antimicrobial usage is regulated by national laws aligned with EU directives, emphasizing prescription-only access and the restriction of critically important antibiotics. However, differences in implementation, enforcement intensity, and farming practices compared to other European countries may influence local AMR trends. For instance, Denmark and the Netherlands have more stringent reduction programs, leading to lower antimicrobial usage rates [68].

The high prevalence of MDR and XDR strains emphasizes the urgency of revising antibiotic policies and exploring alternative therapeutic options. A combination of strategies is necessary to mitigate AMR development in duck farming. These include implementing stricter regulations on antibiotic usage, promoting vaccination programs to reduce infection pressure, improving farm biosecurity measures such as controlled access, sanitation protocols, and all-in/all-out production systems, and enhancing farmer education on responsible antibiotic stewardship. Investment in microbiological monitoring and alternative therapies, such as probiotics and bacteriophage applications, could also play a role in reducing antimicrobial dependency.

## 4. Materials and Methods

### 4.1. Origin of Bacterial Strains and Human Resistance Data

The *E. coli* isolates analyzed in this study were collected between 2022 and 2023 and were isolated by experts at the National Reference Laboratory of the National Food Chain Safety Office in Hungary (NÉBIH) from deceased and diagnostically necropsied ducks; following the guidelines of ISO 16649-2:2001 for the enumeration and isolation of coliform bacteria [69]. The necropsies were performed by poultry health specialists, while the bacterial isolations were carried out by laboratory technicians. The *E. coli* isolates were initially isolated using Coliform agar (Biolab Zrt., Budapest, Hungary) following the ISO 16649-2:2001 protocol. Presumptive identification was based on colony morphology, and isolates were received as pure cultures for further analysis. The isolates were received as pure cultures and stored in Microbank™ cryopreservation vials (Pro-Lab Diagnostics, Richmond Hill, Canada) containing a cryoprotective bead and a proprietary cryopreservative medium at −80 °C. A total of 108 *E. coli* isolates were recovered from ducks, while 58,168 human isolates were analyzed for comparison.

Human antimicrobial resistance data were provided by the National Public Health Center of Hungary. Human resistance data were based on ampicillin resistance, while in veterinary cases, amoxicillin was considered. For third-generation cephalosporins, ceftriaxone resistance was compared between human and animal isolates. Resistance data for aminoglycosides were aggregated for gentamicin, tobramycin, and amikacin, with additional specific data available for neomycin. Similarly, fluoroquinolone resistance was analyzed collectively, while enrofloxacin resistance was examined separately for veterinary isolates. It is important to note that ceftriaxone is not authorized for routine veterinary use. In veterinary medicine, third-generation cephalosporins such as ceftiofur are commonly used, particularly in cattle. For the detection of ESBL-producing strains, cefotaxime or ceftazidime is typically employed as a screening agent in both human and veterinary microbiological practices. The human resistance data, including both national and region-specific statistics, were provided in an Excel file with the approval of the Chief Medical Officer of Hungary. The dataset contained resistance prevalence expressed as percentage values.

For *E. coli* isolates from ducks, information was available on the organ of origin (liver, bone marrow, lungs, brain chamber, articulatio, pericardium) and the geographic location of sample collection. Based on the collection sites, the isolates were categorized into one of Hungary’s seven administrative regions.

### 4.2. Minimum Inhibitory Concentration (MIC) Determination

Phenotypic resistance expression was assessed by determining MICs following the guidelines of the Clinical Laboratory Standards Institute (CLSI) [70]. Breakpoints were established according to CLSI recommendations [71], and results were compared against the ECOFFs defined by the EUCAST [72]. The literature references for MIC determination were consulted, including those for amoxicillin–clavulanate [73], neomycin [74], spectinomycin [73], and colistin [75].

Bacterial strains stored at −80 °C were suspended in 3 mL of cation-adjusted Mueller–Hinton broth (CAMHB) and incubated at 37 °C for 18–24 h before testing. MIC testing was conducted using 96-well microtiter plates (VWR International, LLC, Debrecen, Hungary). Except for the first column, all wells were filled with 90 µL of CAMHB. Stock solutions of tested antimicrobial agents (Merck KGaA, Darmstadt, Germany) at 1024 µg/mL were prepared following CLSI guidelines [71].

Amoxicillin and amoxicillin-clavulanate were dissolved at a 2:1 ratio in phosphate-buffered solution (pH 7.2, 0.01 mol/L), while imipenem was dissolved in phosphate buffer at pH 6.0 (0.1 mol/L). Doxycycline, neomycin, tilozin, and vancomycin were dissolved in distilled water. For potentiated sulfonamides (trimethoprim and sulfamethoxazole at a 1:19 ratio), sulfamethoxazole was dissolved in hot water with a few drops of 2.5 mol/L NaOH, while trimethoprim was dissolved in distilled water containing 0.05 mol/L HCl. Enrofloxacin was prepared using distilled water with a few drops of 1 mol/L NaOH, while florfenicol was dissolved in distilled water with 95% ethanol.

A 1:1 dilution of the 512 µg/mL antimicrobial solutions was prepared in CAMB, and 180 µL was added to the first column of the microtiter plates, followed by two-fold serial dilutions. After the 10th column, excess 90 µL was discarded to ensure uniform volume. Bacterial suspensions were adjusted to 0.5 McFarland standard using a nephelometer (ThermoFisher Scientific, Budapest, Hungary) and inoculated into the microtiter plates from the 11th column backward, at a 10 µL/well concentration [70].

MIC readings were obtained using the Sensititre™ SWIN™ automatic MIC reader (ThermoFisher Scientific, Budapest, Hungary) and analyzed with VIZION system software v3.4 (ThermoFisher Scientific, Budapest, Hungary, 2024). The reference quality control strain was *E. coli* (ATCC 25922).

### 4.3. Statistical Analysis

Statistical analyses were performed using R (v4.2.2) in the RStudio (2023.09.1) environment [76]. The normality of data distribution was tested using the Shapiro–Wilk test. Non-normally distributed data were analyzed using non-parametric tests. Differences in resistance levels between groups were assessed using the Kruskal–Wallis test [77], which does not assume normal distribution and is well-suited for comparing multiple group medians. Post hoc analyses were conducted using the Mann–Whitney U test [78], and *t*-tests, with pairwise comparisons corrected using the Bonferroni correction [79], to control for inflated *p*-values due to multiple testing. It should be noted that Bonferroni correction can increase the likelihood of Type II errors, potentially reducing the ability to detect true differences.

Correlation analysis of antimicrobial resistance among antibiotics was performed using a heatmap analysis, employing the corrplot (v0.92) and pheatmap (v1.0.12) packages.

For cluster analysis, hierarchical clustering was conducted using the factoextra (v1.0.7) package for visualization, cluster (v2.1.4) for agglomerative hierarchical clustering, and dendextend (v1.16.0) for dendrogram visualization.

Network analysis was performed to examine cross-resistance patterns among antibiotics. Graphs were constructed and analyzed using igraph (v1.3.5), while ggraph (v2.1.0) was used for visualization.

For MDR strain prediction, a decision tree model was developed using rpart (v4.1.16). Model performance was evaluated using the caret (v6.0.93) package, and the decision tree was visualized with rpart.plot (v3.1.2).

Monte Carlo simulations were conducted to estimate MDR prevalence under different antibiotic usage scenarios. Bootstrap sampling with 10,000 iterations was performed using the boot (v1.3.28) package, while data aggregation and statistical calculations were carried out using dplyr (v1.1.0). Simulation result distributions were visualized using ggplot2 (v3.4.0).

All analyses were performed using open-source R VERSION 4.2.2 packages, ensuring full reproducibility of the study.

## 5. Conclusions

The spread of antibiotic-resistant bacterial strains poses both veterinary and public health challenges, necessitating the development of new antimicrobial agents and alternative therapeutic approaches. Our findings underscore the growing significance of AMR in waterfowl farming, particularly given the high prevalence of MDR and XDR *E. coli* strains. The patterns of resistance and network analysis results reveal strong cross-resistance relationships among certain antibiotics, which are likely to be influenced by antibiotic usage practices, although this requires further investigation to be conclusively demonstrated.

The application of predictive models, particularly decision trees and neural networks, proved to be effective tools for identifying MDR strains, enhancing our understanding of AMR dynamics and aiding in their prediction. However, it is important to note that while statistical correlations identified by predictive models may provide valuable insights, they do not necessarily imply direct biological causation. Experimental validation is needed to confirm the biological relevance of the observed associations. Additionally, Monte Carlo simulations provided further insights into the potential occurrence of MDR strains, reinforcing the necessity of continuous epidemiological monitoring.

Overall, these findings emphasize the importance of a multidisciplinary approach in combating antimicrobial resistance, integrating ongoing surveillance, data-driven decision-making, and responsible antibiotic stewardship. Future research should focus on gaining a more detailed mechanistic understanding of cross-resistance development and its clinical implications.

## Figures and Tables

**Table 1:** The frequency distribution table of minimum inhibitory concentrations (MICs) for *Escherichia coli* isolates (*n* = 108) from ducks, tested against antibiotics with established clinical breakpoints. The upper row represents the frequency values, while the lower row indicates the corresponding percentage. Vertical red lines denote the clinical breakpoints, while vertical green lines represent the epidemiological cut-off values (ECOFF) defined by the European Committee on Antimicrobial Susceptibility Testing (EUCAST).

Antibiotics	Breakpoint	0.001	0.002	0.004	0.008	0.016	0.031	0.063	0.125	0.25	0.5	1	2	4	8	16	32	64	128	256	512	1024	MIC_50_	MIC_90_	ECOFF ^3^
	(µg/mL)																								
Amoxicillin	32										5	0	4	22	22	5	2	27	3	5	5	8	16	512	8
											4.6%	0.0%	3.7%	20.4%	20.4%	4.6%	1.9%	25.0%	2.8%	4.6%	4.6%	7.4%			
Amoxicillin-clavulanic acid ^1^	32						1	0	0	0	5	1	7	29	25	20	7	10	1	1	1		8	64	8
							0.9%	0.0%	0.0%	0.0%	4.6%	0.9%	6.5%	26.9%	23.1%	18.5%	6.5%	9.3%	0.9%	0.9%	0.9%				
Ceftriaxone	4					5	23	28	7	8	4	6	3	0	2	3	8	3	3	3	1	1	0.06	32	0.125
						4.6%	21.3%	25.9%	6.5%	7.4%	3.7%	5.6%	2.8%	0.0%	1.9%	2.8%	7.4%	2.8%	2.8%	2.8%	0.9%	0.9%			
Colistin	2						3	2	6	28	16	11	5	1	2	1	16	0	2	1	4	10	0.5	512	2
							2.8%	1.9%	5.6%	25.9%	14.8%	10.2%	4.6%	0.9%	1.9%	0.9%	14.8%	0.0%	1.9%	0.9%	3.7%	9.3%			
Doxycycline	16									1	2	6	33	13	12	10	18	7	6				4	64	8
										0.9%	1.9%	5.6%	30.6%	12.0%	11.1%	9.3%	16.7%	6.5%	5.6%						
Enrofloxacin	2					3	22	6	7	3	22	7	3	4	2	4	14	2	8	0	1		0.5	32	0.125
						2.8%	20.4%	5.6%	6.5%	2.8%	20.4%	6.5%	2.8%	3.7%	1.9%	3.7%	13.0%	1.9%	7.4%	0.0%	0.9%				
Florfenicol	16												2	10	33	34	13	3	10	2	1		16	128	16
													1.9%	9.3%	30.6%	31.5%	12.0%	2.8%	9.3%	1.9%	0.9%				
Imipenem	4				1	0	1	4	8	21	26	28	15	1	2	1							0.5	16	0.5
					0.9%	0.0%	0.9%	3.7%	7.4%	19.4%	24.1%	25.9%	13.9%	0.9%	1.9%	0.9%									
Neomycin	32													1	2	9	39	43	5	1	6	2	64	128	8
														0.9%	1.9%	8.3%	36.1%	39.8%	4.6%	0.9%	5.6%	1.9%			
Potentiated sulphonamide ^2^	4											4	14	21	12	12	4	2	2	19	5	13	16	1024	0.5
												3.7%	13.0%	19.4%	11.1%	11.1%	3.7%	1.9%	1.9%	17.6%	4.6%	12.0%			
Spectinomycin	128													1	0	0	23	69	8	4	0	3	64	128	64
														0.9%	0.0%	0.0%	21.3%	63.9%	7.4%	3.7%	0.0%	2.8%			

^1^ ratio 2:1; ^2^ trimethoprim and sulfamethoxazole in a 19:1 ratio, ^3^ Epidemiological cut-off values (ECOFF) defined by the European Committee on Antimicrobial Susceptibility Testing (EUCAST).

## Data Availability

The data presented in this study are available from the corresponding author upon reasonable request.

## Supplementary Materials

The following supporting information can be downloaded at: https://www.mdpi.com/article/10.3390/antibiotics14050491/s1. Figure S1: Proportion of non-wild-type strains per antimicrobial agent based on the epidemiological cut-off values (ECOFF) defined by the European Committee on Antimicrobial Susceptibility Testing (EUCAST). Table S1: Frequency distribution table of minimum inhibitory concentrations (MICs) for *Escherichia coli* isolates (*n* = 108) from ducks, tested against antibiotics without established clinical breakpoints. The upper row represents the frequency values, while the lower row indicates the corresponding percentage.

## Abbreviations

The following abbreviations are used in this manuscript:

AMR	Antimicrobial resistance
CAMHB	Cation-adjusted Mueller Hinton Broth
CLSI	Clinical Laboratory Standards Institute
ECOFF	Epidemiological cut-off values
ESBL	Extended-spectrum beta-lactamases
EUCAST	European Committee on Antimicrobial Susceptibility Testing
MDR	Multidrug-resistant
MIC	Minimum inhibitory concentration
WHO	World Health Organization
XDR	Extensively drug-resistant
