# Supplementary material for: Antimicrobial Susceptibility Profiles of Escherichia coli Isolates from Clinical Cases of Ducks in Hungary Between 2022 and 2023
Source: Antibiotics (Basel). 2025 May 10;14(5):491. doi: 10.3390/antibiotics14050491 (PMC12108305; doi:10.3390/antibiotics14050491)
Supplement: Supplementary file 1 [file antibiotics-14-00491-s001.zip › Supplementary Materials.pdf]

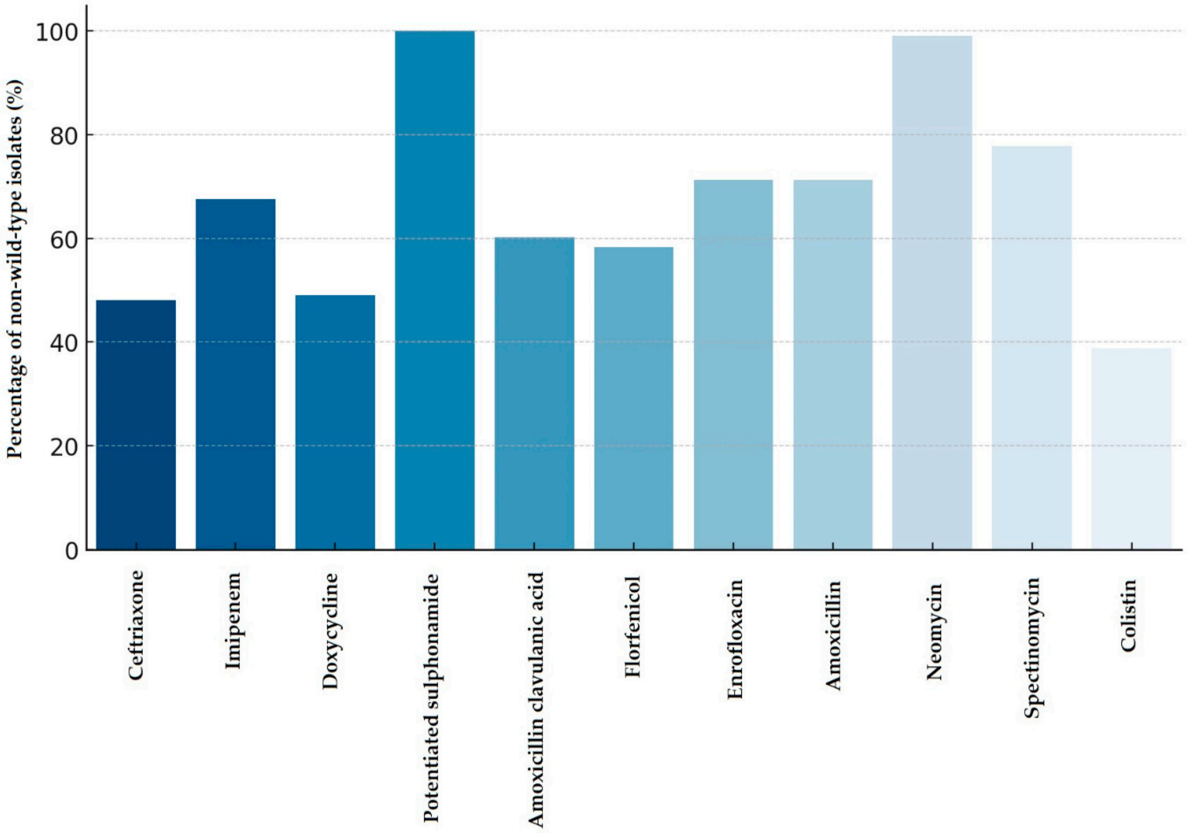

Supplementary Figure S1 Proportion of non-wild-type strains per antimicrobial agent based on the epidemiological cut-off values (ECOFF) defined by the European Committee on Antimicrobial Susceptibility Testing (EUCAST).

Supplementary Table S1 Frequency distribution table of minimum inhibitory concentrations (MICs) for *Escherichia coli* isolates (*n* = 108) from ducks, tested against antibiotics without established clinical breakpoints. The upper row represents the frequency values, while the lower row indicates the corresponding percentage.

**Antibiotics**	**0.001**	**0.002**	**0.004**	**0.008**	**0.016**	**0.031**	**0.063**	**0.125**	**0.25**	**0.5**	**1**	**2**	**4**	**8**	**16**	**32**	**64**	**128**	**256**	**512**	**1024**	**MIC$_{50}$**	**MIC$_{90}$**
	**(µg/mL)**																						
Lincomycin												1	0	0	0	0	1	63	0	7	36	128	1024
												0.9%	0.0%	0.0%	0.0%	0.0%	0.9%	58.3%	0.0%	6.5%	33.3%		
Tiamulin																	3	74	11	10	10	128	512
																	2.8%	68.5%	10.2%	9.3%	9.3%		
Tilozin																2	0	65	2	20	19	128	1024
																1.9%	0.0%	60.2%	1.9%	18.5%	17.6%		
Vancomycin																	2	0	20	82	4	512	512
																	1.9%	0.0%	18.5%	75.9%	3.7%		
